# Tracking microhabitat temperature variation with iButton data loggers

**DOI:** 10.1002/aps3.1237

**Published:** 2019-04-08

**Authors:** Susan Fawcett, Seeta Sistla, Manny Dacosta‐Calheiros, Abdullah Kahraman, Anton A. Reznicek, Rachel Rosenberg, Eric J. B. von Wettberg

**Affiliations:** ^1^ Pringle Herbarium, Department of Plant Biology University of Vermont 63 Carrigan Drive Burlington Vermont 05401 USA; ^2^ Biological and Life Sciences Hampshire College 893 West Street Amherst Massachusetts 01002 USA; ^3^ Biological Sciences Florida International University 11200 SW 8th Street (CP‐304) Miami Florida 33199 USA; ^4^ Department of Field Crops Harran University Osmanbey Yerleşkesi Şanlıurfa‐Mardin Karayolu Üzeri 18 Km 63300 Şanlıurfa Turkey; ^5^ University of Michigan Herbarium 3600 Varsity Drive Ann Arbor Michigan 48108 USA; ^6^ Department of Plant and Soil Science University of Vermont 63 Carrigan Drive Burlington Vermont 05401 USA

**Keywords:** crop wild relatives, fern ecology, flowering time, microclimate, niche modeling, phenology, solar

## Abstract

**Premise of the Study:**

Fine‐scale variation in temperature and soil moisture contribute to microhabitats across the landscape, affecting plant phenology, distribution, and fitness. The recent availability of compact and inexpensive temperature and humidity data loggers such as iButtons has facilitated research on microclimates.

**Methods and Results:**

Here, we highlight the use of iButtons in three distinct settings: comparisons of empirical data to modeled climate data for rare rock ferns in the genus *Asplenium* in eastern North America; generation of fine‐scale data to predict flowering time and vernalization responsiveness of crop wild relatives of chickpea from southeastern Anatolia; and measurements of extreme thermal variation of solar array installations in Vermont.

**Discussion:**

We highlight a range of challenges with iButtons, including serious limitations of the Hygrochron function that affect their utility for measuring soil moisture, and methods for protecting them from the elements and from human interference. Finally, we provide MATLAB code to facilitate the processing of raw iButton data.

Fine‐scale variation in temperature and humidity has major implications for plant phenology, distribution, and fitness but is difficult to capture using climate models. Until relatively recently, obtaining empirical data that demonstrate climatic heterogeneity at fine scales across the landscape required the deployment of costly and conspicuous equipment (e.g., Wethey, 2002; Lundquist and Lott, 2008), inhibiting long‐term or widespread deployment. The availability of affordable, compact, self‐contained temperature and humidity data loggers such as iButtons (Maxim Integrated, San Jose, California, USA) has enabled biologists to generate empirical data of unprecedented quantity and quality. Although such sensors have been used in field biology applications for more than two decades, opportunities and challenges remain to effectively use them.

This technology provides opportunities to develop a more nuanced understanding of the factors underlying plant phenology, such as the timing and temperature requirements for vernalization and flowering time, which have important applications in agriculture and primary productivity more generally. Furthermore, microclimatic conditions (climate conditions of a scale as fine as 1 m, particularly when the surrounding area has a different climate) influence biogeochemical dynamics such as decomposition, which governs plant‐available nutrients (Todd‐Brown et al., 2012). Another valuable use for data loggers is to identify and characterize microclimates that may serve as important refugia through space and time for rare or endangered species. Ultimately, the incorporation of microclimate data can serve a valuable role in validating and improving increasingly complex climate models (Botkin et al., 2007; Dobrowski, 2011; Lembrechts et al., 2018).

The use of correlative species distribution models is the most prevalent means of predicting the impacts of climate change on biodiversity (Franklin et al., 2013). These models typically apply a climate envelope to existing species distribution, i.e., climatic niche, with the prediction that the species will track that envelope through space and time, most commonly upslope or pole‐ward under warming conditions (Moritz and Agudo, 2013). In addition to predicting the future range of species of conservation concern, they are used to evaluate the potential for the expansion of invasive species (Jeschke and Strayer, 2008). Climate envelope models have been used to assess potential invasiveness and to screen plant introductions in Australia, New Zealand, the Galápagos Islands, and Hawaiian Islands (Pheloung et al., 1999; Rogg et al., 2005; Daehler et al., 2004; but see Mandle et al., 2010).

A fundamental criticism of this approach is the lack of understanding of underlying variables and limiting factors responsible for current or future species distributions and the scales at which they are biologically relevant, as well as the inherent uncertainty involved in extrapolation (Pearson and Dawson, 2003; Williams and Jackson, 2007). Models may overestimate risk of extinction because they do not account for heterogeneity of climate and warming rates at the landscape scale, and fail to identify potential climate refugia (Ashcroft, 2010).

Correlative predictive models may be well suited to characterizing climatic niche in certain contexts, in particular across homogeneous landscapes where atmospheric temperature is closely correlated with surface conditions. However, most models rely on coarse‐scale resolution, rarely <1 km, and fail to capture microclimates that vary on a scale of tens to hundreds of meters or less, especially across topographically complex landscapes (Sears et al., 2011; Moritz and Agudo, 2013; Lembrechts et al., 2018). The scale of the species distribution model itself may have a major influence on projected range, with coarser scales potentially overestimating habitat. Franklin et al. (2013) found that a larger‐scale model (4 km^2^) overestimated stable species habitat by an average of 42% compared to a finer‐scale model (800 m) in a comparison of 52 California plant species, and that species with narrow ranges showed the greatest incongruity of predicted ranges between grid sizes. The discrepancy between predictions in areas of extreme topographic heterogeneity may be even greater. Randin et al. (2009) predicted a total elimination of suitable alpine habitat during the 21st century applying a 16‐km grid, in contrast to the persistence of as much as 100% of habitat when analyzed with a 25‐m grid, highlighting the importance of fine‐scale landscape heterogeneity and microclimate in buffering the effects of changing climate (Willis and Bagwhat, 2009; Ackerly et al., 2010; Lenoir et al., 2013).

Microclimates result from the interaction between regional advective influences and local terrain (e.g., Dobrowski, 2011). Within an area of 16 km^2^ (a typical grid size employed by climate models), temperature may vary by as much as 33°C (Hijmans et al., 2005). The primary topographic features responsible for this variation are: elevation, valleys or basins, slope, and aspect, which manifest as local differences in precipitation, wind, insolation, cold air drainage, evapotranspiration, snowmelt, and accumulation (Dobrowski, 2011). In addition to topography, the effects of prevailing winds, proximity to water, and zones of high relative water availability, including rock outcrops, seeps, fog belts, and canopy cover, also contribute to microclimates (Ashcroft et al., 2010; De Frenne et al., 2013; McLaughlin et al., 2017). The spatiotemporal scale at which climate is biologically important will differ according to taxon, but for sessile organisms like plants, fine‐scale differences are important (Lembrechts et al., 2018). Because microclimates are often loosely coupled to regional climate, they may serve as important buffers to climate change. Identifying microclimates on the landscape and understanding how they are utilized by the species occurring within them are essential to project the impacts of climate change. Based on fossil evidence, microclimates may have served as important refugia during past periods of climate change (Petit et al., 2008; Hof et al., 2011; Willis and MacDonald, 2011; Moritz and Agudo, 2013), and understanding their conservation potential should be a research priority (Keppel et al., 2012, 2015).

Understanding the extent of adaptation to microclimates is also potentially important to harnessing crop wild relatives in breeding programs (e.g., Warschefsky et al., 2014). For example, crop wild relatives from 1‐km grid cells with overall arid conditions may mislead researchers using methods like the Focused Identification of Germplasm Strategy (FIGS; Khazaei et al., 2013) into believing they hold drought‐adapted ecotypes if local populations are restricted to humid or mesic microsites. The finer‐scale information provided by iButtons can supplement such models and provide greater insight into microsite preferences.

Effective conservation strategies also depend on understanding microclimate variation. Many recent coarse‐scale models predict catastrophic loss of climatic niches under warming scenarios (e.g., Thomas et al., 2004; Dullinger et al., 2012). In stark contrast, Scherrer and Körner (2011) demonstrated remarkable buffering capacity provided by topographically induced microclimates in the Swiss Alps, using empirical data recorded using iButton data loggers to measure soil temperature. Within an area of 2 km^2^, the recorded mean annual temperatures in the soil differed by as much as 10.5°C from air temperatures. Based on their measurements, under a 2°C warming scenario (Pachauri et al., 2014), only the coolest 3% of habitat will be lost, and the migration distances required for species to track their current climatic envelope are within meters. In this alpine study site, meter‐scale soil temperature variation is greater than the extent of expected warming in Intergovernmental Panel on Climate Change projections for the next 100 years.

Consequently, more precise methods can improve our capacity to predict the distribution and phenology of species that may depend on microclimates. Data loggers such as iButtons have the capacity to measure temperature at desired intervals, generating precise data for conditions aboveground, at the soil surface, and underground. These data can be useful for developing phenological models, which can better account for the mechanisms by which plant emergence, flowering, and senescence may need to shift to account for varying climatic conditions. Plant phenological models take many forms, with a number of variants being widespread in the agricultural literature (e.g., Boote et al., 1998; Jones et al., 2003) and the *Arabidopsis* literature (e.g., Wilczek et al., 2009; Chew et al., 2012), where the extensive detail needed to parameterize such models is available. Photothermal models like that of Chew et al. (2012) account for both day length and temperature, as well as vernalization, incorporating the effect of genetic variants in key pathways affecting phenology. The inclusion of precise temperature data from sites of origin improves the accuracy of these models.

## METHODS AND RESULTS

### Three case studies using iButton data loggers

We present three case studies representing different applications of fine‐scale temperature measurements for studying the effects of microclimates in plant biology. The first study compares empirically derived iButton data to modeled PRISM and WorldClim climate data for six temperature variables to illustrate fine‐scale microclimatic conditions of a habitat specialist that are not captured by a 1‐km or 4‐km grid, to illustrate the scale of discrepancy that may be anticipated from different approaches. The second case examines the effect of microclimate on growth conditions to predict flowering time and vernalization responsiveness in two crop wild relatives, which provide a basis for parameterizing phenological models. The third example demonstrates microhabitat gradients caused by the built environment, highlighting the complex dynamics of soil temperature and moisture resulting from solar installations in a snowy northern climate. We highlight these examples in the context of the diverse applications of iButtons in plant biology, and discuss important experimental considerations and limitations of the technology and strategies for addressing them. Finally, we provide MATLAB code for processing and analyzing raw iButton data, which is useful when large numbers of loggers have been used.

#### Case study 1: A comparison of empirical and modeled temperature data for rare rock fern habitats

**Figure 1:**
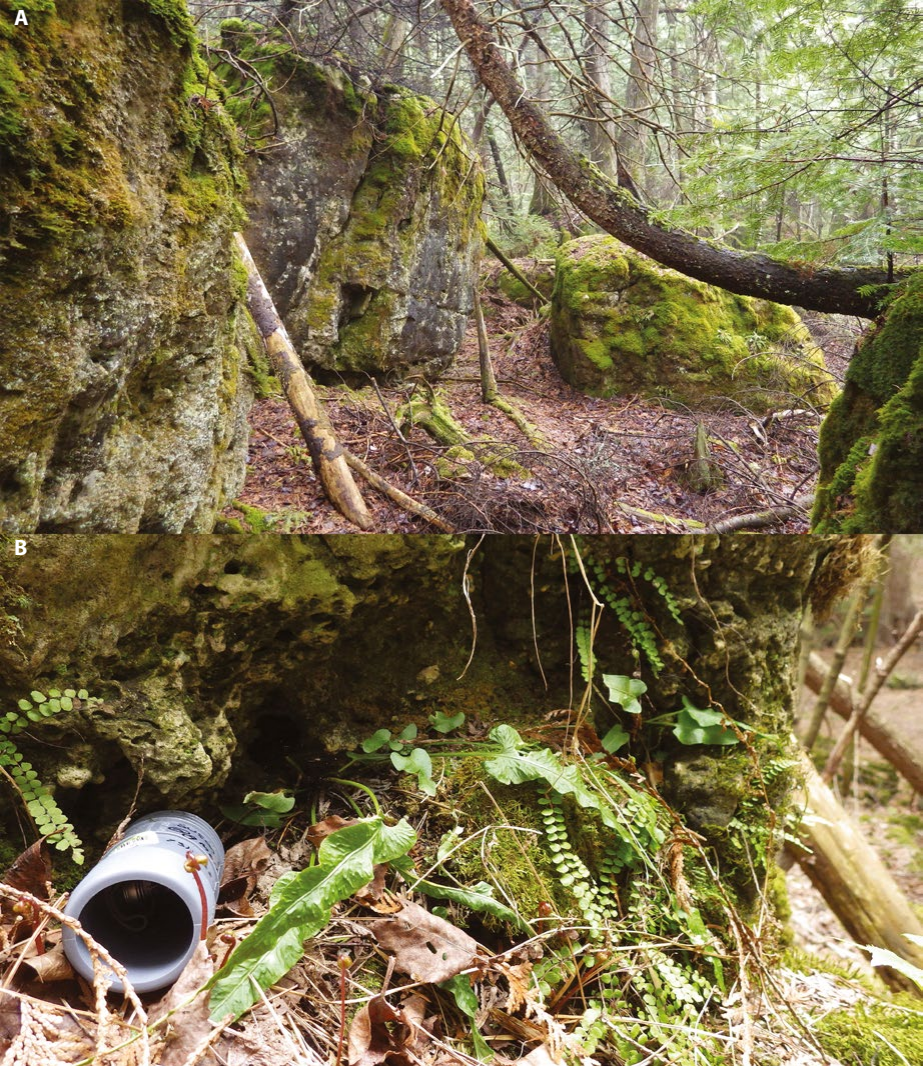
(A) Typical habitat supporting both *Asplenium viride* and *A. rhizophyllum*, showing limestone boulders associated with the Niagara Escarpment in Michigan's eastern Upper Peninsula. (B) PVC pipe with iButton Hygrochron mounted inside, placed in close proximity to *A. rhizophyllum*.

We examine microhabitat conditions of two narrowly restricted rock ferns, *Asplenium viride* Huds. and *A. rhizophyllum* L., to highlight the discrepancy between modeled climate and empirical measurements for these habitat specialists. Both species are calciphilic rock ferns and occur predominantly on limestone, in shaded understories (Fig. 1A). In addition to edaphic specialization, their habitats often coincide with topographical extremes on both macro and micro scales (sensu Ackerly et al., 2010). For example, they are sensitive to slope and aspect at the scale of both decameters and centimeters, predominantly occupying north‐ and east‐facing slopes. In addition to requiring the microtopography provided by rock outcrops or similar terrains that are typically regionally restricted, their habitats often represent mesic microsites (sensu McLaughlin et al., 2017). These ferns achieve most luxuriant growth in proximity to the moderating influence of Great Lakes shorelines, inland lakes, conifer swamps, streamsides, mountain peaks, sinkholes, glacial cirques, and deep canyons. Finally, these ferns occur almost exclusively under dense canopy cover, which is crucial to maintaining high relative humidity (Chen et al., 1999; Lendzion and Leuschner, 2009) and has been shown to be more important than air temperature or soil moisture for the growth of *Polystichum braunii* (Spenn.) Fée, another rare fern of calcareous substrates that frequently co‐occurs with *A. viride* and *A. rhizophyllum* (Schwerbrock and Leuschner, 2016).

To measure temperature in the fern microsites, 37 Maxim Integrated DS1923 iButton Hygrochron temperature/humidity loggers were deployed in close proximity to *A. viride* and *A. rhizophyllum* at sites in the U.S. states of Wisconsin, Michigan, New York, and Vermont, and the Canadian province of Ontario, in the spring and summer of 2016, and retrieved approximately one year later. The iButtons were set to record temperature and humidity every 4 h for one year with no rollover, and mounted inside a small section of PVC pipe to protect them from direct precipitation, while not obstructing the humidity aperture. A neutral gray‐colored PVC pipe was chosen because it was less conspicuous in the environment than white. To capture the fern microsite conditions, the pipes were placed as close to the ferns as possible, usually within centimeters, typically wedged into mossy crevices of limestone boulders (Fig. 1B). The coordinates of the iButtons and associated research plots were recorded with a GPS unit, and each site was photographed extensively, while the precise location of the iButton was sketched and described in field notes. To facilitate relocation, an iPad in a waterproof case was taken into the field to display the photographs. Upon retrieval of the iButtons, data were downloaded using the DS1402D‐DR8 Maxim Integrated Blue Dot Receptor.

Of the 37 iButtons placed in the fern microsites, 31 were recovered, but only 11 yielded data, representing sites in Wisconsin, Michigan, Ontario, and Vermont. For the six not recovered, the precise locations were relocated with confidence based on the photographs. The remaining 20 iButtons either were blank and did not register on the receptor, or else had an error message, and were likely compromised by extremes in temperature and humidity.

Modeled data from two sources were compared to the iButton data. PRISM provides data of 4‐km resolution that can be extrapolated to correspond to the exact dates of iButton deployment, whereas WorldClim data provide 1‐km resolution, but are based on recent multi‐year means. Using GPS coordinates from iButton locations, PRISM data were downloaded from the PRISM Climate Group (Oregon State University; http://www.prism.oregonstate.edu), and WorldClim Bioclimatic variables (Fick and Hijmans, 2017) were downloaded using the R package *raster* version 2.6‐7 (Hijmans and van Etten, 2012). These data were then compared to the empirical data recorded by the iButtons using a paired *t*‐test. Because climate data were only available for the conterminous United States, the PRISM comparison was restricted to eight U.S. sites. In some cases, the iButtons were retrieved slightly before the full 365 days, in which case the monthly mean value was used in place of the missing data. Comparisons were made for mean annual temperature, maximum temperature of warmest month, minimum temperature of coldest month, annual range in temperature, mean temperature of warmest quarter, and mean temperature of coldest quarter. Boxplots were generated in R.

**Figure 2:**
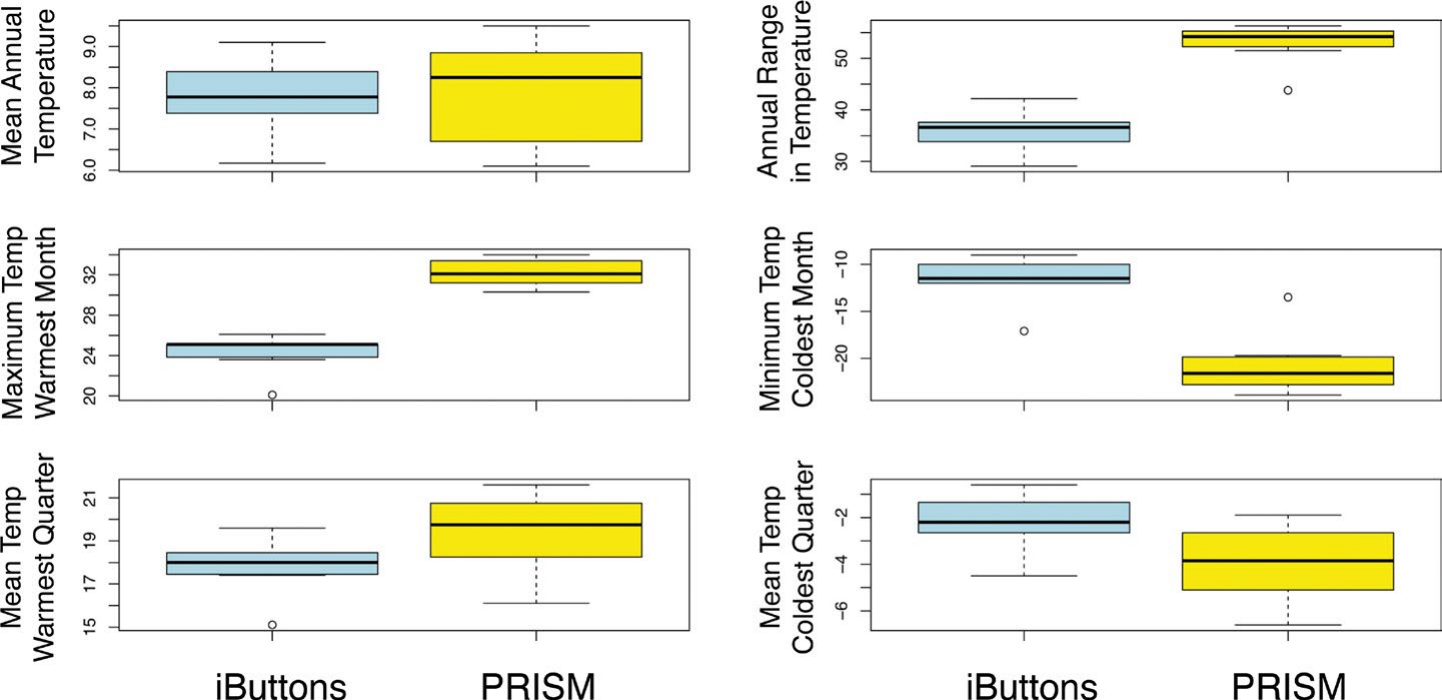
A comparison between one year (2016–2017) of empirically derived temperatures from iButton data loggers and modeled temperatures from PRISM extrapolations at 4‐km resolution for eight microhabitats in Wisconsin, Michigan, and Vermont. Mean annual temperature: *t* = 0.41, *df* = 7, *P* = 0.70; mean annual range in temperature: *t* = 7.82, *df* = 7, *P* = 0.0001; maximum temperature of warmest month: *t* = 9.73, df = 7, *P* = 0.00003; minimum temperature of coldest month: *t* = −6.23, *df* = 7, *P* = 0.0004; mean temperature of warmest quarter: *t* = 2.79, *df* = 7, *P* = 0.02; mean temperature of coldest quarter: *t* = −2.90, *df* = 7, *P* = 0.02. Temperatures are in °C.

A comparison of empirical data to modeled data demonstrates that the microhabitats occupied by these ferns buffer extremes in temperature (Fig. 2). All comparisons with PRISM were significant when α = 0.05, except for mean annual temperature. The strongest differences are seen in minimum temperature of coldest month, where microclimates average 9.1°C warmer than the model (*P* = 0.0004), and maximum temperature of warmest month, where average microclimates average 7.9°C cooler (*P* = 0.00003). The minima and maxima may be influenced somewhat by the 4‐h sampling interval of the iButtons, however, the pattern is upheld by the quarterly means, with mean temperature of the coldest quarter 1.6°C warmer (*P* = 0.02) and mean temperature of the warmest quarter 1.8°C cooler (*P* = 0.02) for iButton sites.

The greatest difference between the iButton data and the WorldClim data (not shown) was for mean temperature of the coldest quarter, with the measured sites 5.3°C warmer than the model (*t* = −9.68, *df* = 10, *P* < 0.00001). Additionally, iButton measurements for mean annual temperature were 1.8°C warmer (*t* = −7.6, *df* = 10, *P* < 0.00001). Annual range in temperature was 3.8°C greater for WorldClim (*t* = 2.22, *df* = 10, *P* = 0.05), whereas maximum temperature of warmest month (*P* = 0.07), minimum temperature of coldest month (*P* = 0.07), and mean temperature of warmest quarter (*P* = 0.23) were not significantly different. Although the WorldClim data represent a finer scale (1 km) than the PRISM data (4 km), they reflect historic means, rather than the exact time period available from PRISM, and are therefore less appropriate for predicting current climate.

Although the iButtons were distributed more or less equally between *A. viride* and *A. rhizophyllum* sites, five of the six that were not recovered were at *A. viride* sites. We noted that *A. viride* frequently grows in close proximity to water courses, and animal interference is suspected in at least three cases. On other cases, collapsing talus slopes, violent spring runoff, or ice‐fall may have resulted in iButton loss. In the case of the single iButton lost from an *A. rhizophyllum* site, based on our observations, we believe deer may have disturbed the site while scraping moss and ferns from the boulder. There was also evidence of recent human disturbance in the area, which is another possibility. In one case, a hiker retrieved a displaced iButton and contacted us using the information on the iButton housing. Of the 11 iButtons that yielded data, only three represent *A. viride* sites. The microsites occupied by this species often represented the most extreme environments, i.e., those that were coolest, most humid, at the highest elevations, or with the steepest terrain. The nature of these environments may have played a role in their disproportionate disappearance and failure.

Our results indicate that both correlative species distribution models (i.e., based on a 4‐km resolution corresponding to exact dates and a 1‐km resolution corresponding to recent historical means) fail to represent the climatic niche of the *Asplenium* spp. This comparison demonstrates that the locations of the ferns represented microclimates that were buffered against both the highest and lowest temperature extremes.

#### Case study 2: Microclimatic influences on the phenology of a crop wild relative: *Cicer* (chickpeas)

The microclimatic conditions of *Cicer reticulatum* Ladizinsky—the immediate wild progenitor of cultivated chickpea, *C. arietinum* L., as well a sister species, *C. echinospermum* P. H. Davis—were studied to develop an understanding of the conditions underlying growth and phenology. *Cicer reticulatum* and *C. echinospermum* occur in savannas and pastures in southeastern Turkey (Toker et al., 2014). Microsites can vary from disturbed field edges to rock crevices on mountainsides. In general, *C. echinospermum* occurs in lower‐elevation sites on basaltic soils, whereas *C. reticulatum* occurs at slightly higher elevations and higher pH sandstone‐ or limestone‐derived soils. With a wide range of possible habitats in steep terrain, interpolated climates are not likely to accurately predict microclimatic conditions.

**Figure 3:**
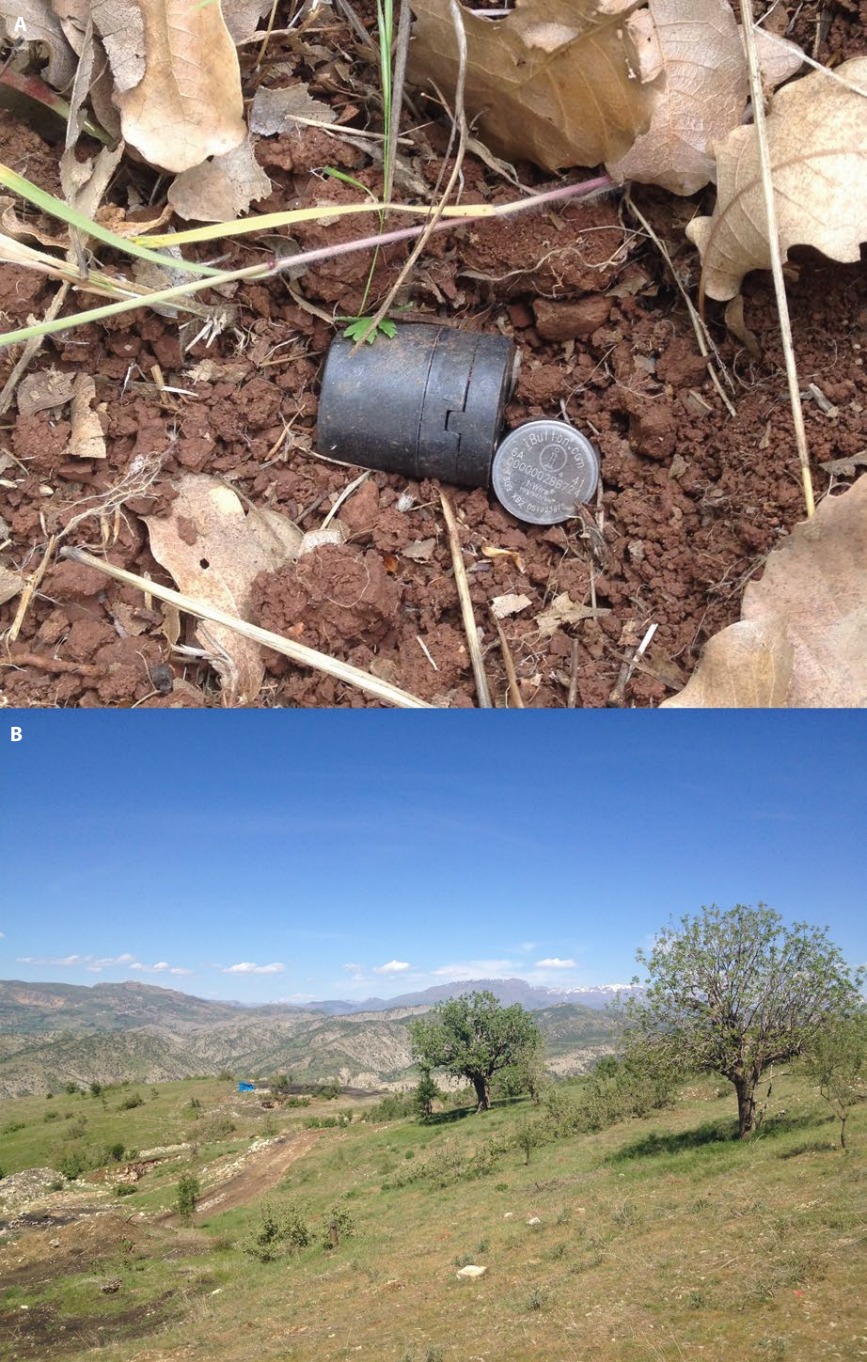
(A) Placement of iButton Hygrochrons and Thermochrons (within black iButton capsules) into soils 5 cm below the surface near *Cicer* plants. (B) Typical wild *Cicer* habitat in southeastern Turkey (from the Cudi habitat in von Wettberg et al., 2018).

Seventeen populations of wild chickpea were chosen to study variation in microsite conditions. For each population (described in von Wettberg et al., 2018), five Maxim Integrated DS1921 iButton Thermochron temperature loggers and five Maxim Integrated DS1923 iButton Hygrochron temperature/humidity loggers were deployed at a depth of 5 cm below the soil surface at random, with a total sample of 170 data loggers placed at the study site (Fig. 3B). Thermochrons were protected by iButton waterproof capsules (Maxim Integrated; Fig. 3A) to prevent exposure to soil moisture. Hygrochrons were placed in the soil without protection. Initial placement in field sites occurred in October 2013. To aid in recovery, GPS coordinates and digital photographs were taken, and small rock cairns near the site or spray‐painted markings on nearby rocks were made. Sensors were first retrieved at the end of May 2014 and returned to the soil after data were downloaded. They were excavated again in October 2014 at four sites. Although initially placed with hopes of further recovery, the declining security situation in southeastern Turkey and neighboring Syria meant we did not attempt to further recover the data loggers. Upon retrieval of the iButtons in May and October 2014, data were downloaded using the DS1402D‐DR8 Maxim Integrated Blue Dot Receptor. Data were processed with custom MATLAB code (available on GitHub: https://github.com/ericvonwettberg/iButtons) due to the large number of files involved. An annotated user manual is also available on GitHub.

A primary aim was to use microsite estimates of growth conditions to predict flowering time and vernalization responsiveness. Flowering time is a key trait adapting chickpea varieties to different climatic zones (Berger et al., 2004, 2006). We converted temperatures to modified photothermal units, following Chew et al. (2012) and as reported in von Wettberg et al. (2018). Although the wild *Cicer* L. species closely related to chickpea have been thought to be vernalization responsive like other Mediterranean winter annuals, loss of vernalization is thought to have been selected to allow chickpea to be grown as a spring annual (Abbo et al., 2003). However, uncovering variation in vernalization sensitivity in wild *Cicer* could allow for the expanded use of fall‐planted chickpea (Pinhasi van‐Oss et al., 2016) and facilitate rapid introgression of wild germplasm (von Wettberg et al., 2018). We assessed the relationship between coldest temperature measured with iButtons and the average thermal minima from the WorldClim Version 1 data set (averages from 1960–1990) with Pearson correlation.

We also deployed iButton Thermochrons and Hygrochrons as we amplified seeds of the wild *Cicer* collection in common garden settings. These data were intended to help calibrate models of flowering time variation (following Chew et al., 2012) and to validate measurements from Turkish sites in more controlled settings. We deployed sensors in outdoor wild *Cicer* plantings at the University of California, Davis, the primary site of seed amplification, as well as the USDA Spillman Farm in Pullman, Washington (four sensors in air and soil), and at four sites in South Florida (a private farm, January to May 2014 [nine sensors]; USDA Chapman Field, October 2015 to February 2016 [two sensors; air and soil]; Pinecrest School, November 2015 to February 2016 [two sensors; air and soil]; Fairchild Tropical Botanic Garden, December 2015 to March 2016 [six sensors; air and soil]). In the first Florida planting, we paired the iButton sensor measurements with soil volumetric water content and temperature measurements from a Decagon EM50 soil moisture sensor (Decagon Devices, Pullman, Washington, USA [now Meter Co.]).

We were able to recover 126 loggers of the 170 placed in the field in Turkey in May 2014. Recovery varied substantially between sites. Although not assessed, our recovery appeared to be substantially lower in sites with shallower soils, where the initial burial was more difficult to perform. This may be due to the soil depth, or inability of one of us (E.J.B.v.W.) to distinguish rock cairns from natural rock piles. At three sites (Kalkan, Egil, Karabache), construction of dirt roads (a preliminary step toward converting pastures to fields that can be plowed and used for irrigated maize or cotton) led to bulldozing of the habitat and the loss of the sensors. In the highest‐elevation site, the loggers were recovered from the soil surface, presumably due to the action of frost.

Modified photothermal units have been previously reported from this data set (von Wettberg et al., 2018), and flowering time models are being developed to explore associations of particular single nucleotide variants that segregate in the wild populations with phenology in common garden settings (Kozlov et al., 2019). We did not detect a significant correlation between the iButton temperature minima and temperature minima from 1960–1990 in the field in Turkey (*r* = 0.32, *P* = 0.22), likely due to microsite effects such as the canopy cover, slope, and aspects of sites, as well as water availability and soil water‐holding capacity during the growing season. The lack of correlation suggests that site of origin data may not accurately predict potential variation in thresholds in temperature regimes required for vernalization. We did detect a significant association between Thermochron temperature and Decagon EM50 temperature (*r* = 0.866, *P* < 0.0001) in common garden settings in the United States. No association was detected between Hygrochron relative humidity and soil volumetric water content (*r* = 0.128, *P* = 0.172) in our common garden settings.

#### Case study 3: Microhabitat effects of land‐based, utility‐scale solar arrays

**Figure 4:**
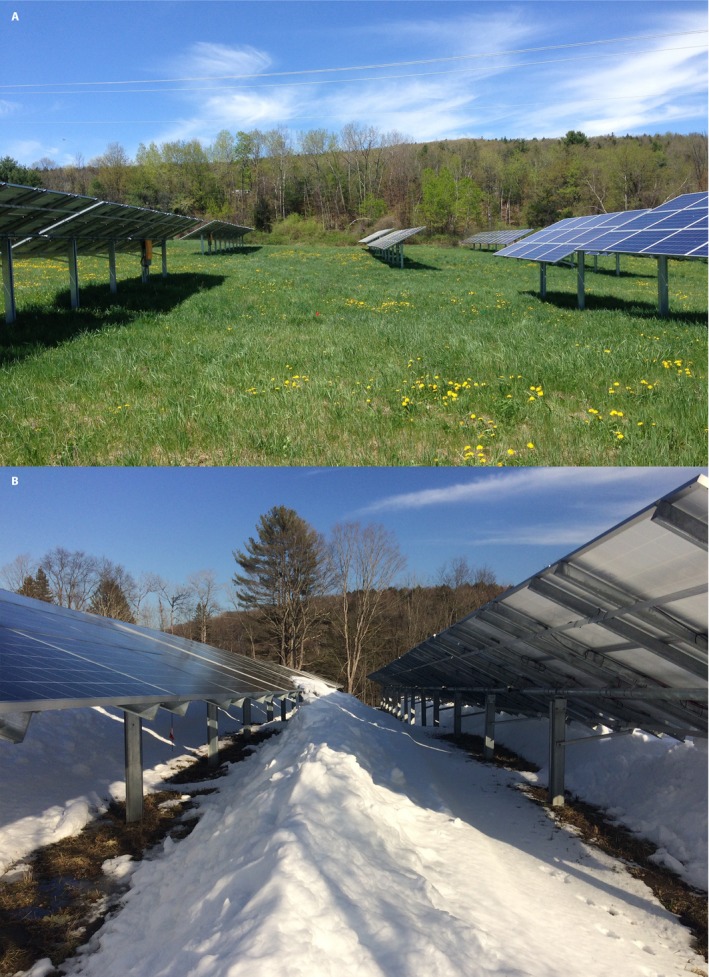
Images of land‐based photovoltaic arrays in the summer (A) and winter (B) in Vermont, USA. The array panels cause a heterogeneous shading and precipitation regime that, over the two decades or more lifespan of the arrays, may drive the formation of persistent novel microhabitats.

Land‐based utility‐scale solar energy (typically ≥1 MW, with ~2 ha of land disturbance per megawatt) ranks among the renewable energy systems with the greatest potential to mitigate climate change (Hernandez et al., 2015). The contribution of solar energy to global power production is rapidly growing, and photovoltaic (PV) arrays have been identified as a change in land use that can merge carbon‐neutral energy generation with habitat conservation or improvement (Armstrong et al., 2014; Hernandez et al., 2015). The placement of PV arrays creates visible differences in precipitation inputs and shading that vary seasonally (Fig. 4). Regular spacing between PV array panels can create a heterogeneous microclimatic regime (Armstrong et al., 2016) that may promote the formation of a novel patchy resource and organismal gradients. For example, PV arrays in the southwestern United States have been found to generate strong heat island effect during the evening (Barron‐Gafford et al., 2016). However, our understanding of how land‐based utility‐scale solar energy arrays impact surface microclimatic features and plant–soil interactions remains limited (Armstrong et al., 2014, 2016; Hernandez et al., 2014).

We studied three PV arrays designed to be community solar providers in Windham County, Vermont, USA, from May 2016 to April 2017 (Sistla et al., unpublished data). Each site varied in soil characteristics, plant community composition, and land use history. The array panels were south facing and arranged in east–west rows with panel height ranging from 1.5 to 2.5 m and site age ranging from 1–3 years old. To determine how PV arrays affect terrestrial microclimate in the temperate zone, we used a haphazardly selected block design consisting of two 1.5‐m^2^ plots, one centered under the PV panel (U) and one centered in the panel‐free row adjacent (A) in each of the three sites at the Vermont Agricultural Center. iButton Thermochrons (DS1921G‐F5#) were used to measure soil temperature, and iButton Hygrochron temperature/humidity loggers (DS1923) measured air temperature and humidity at 1‐h intervals. One Hygrochron was suspended 40 cm above the ground in netting within a radiation shield in the A and U blocks that hosted a micrometeorological station at each site (three U/A Hygrochron pairs consisting of six sensors), which also measured photosynthetically active radiation (PAR) using sensors mounted at 1.2 m (Onset Computer Corporation, Bourne, Massachusetts, USA). Thermochrons measuring soil temperature were protected in 15‐mL conical centrifuge tubes to facilitate retrieval and protect the sensors from moisture and were buried 10 cm deep in both the U and A plots with the weather station, as well as in an additional U/A plot pair haphazardly selected at each site (six U/A Thermochron pairs consisting of 12 sensors).

Data were periodically downloaded on site throughout the study period. All but one of the iButtons (11 Thermochrons and six Hygrochrons) were retrieved after 11 months deployment; none of these Thermochrons appeared to be significantly shifted in the soil profile. One Thermochron could not be located during the January data download and was not subsequently found. Gaps in the data set reflect field deployment errors that were corrected at the following site visit.

Microclimate factors (soil temperature, air temperature, and relative humidity) were separated by site and season (spring [2016], summer [2016], fall [2016], winter [2016–2017], spring [2017]) and further split into day and night, and outliers (defined as any point more than 1.5 times the interquartile range above the third quartile or below the first quartile) were identified to check for the potential for disturbance of the data loggers (i.e., snow accumulation on the Hygrochron sensors). Removal of outliers did not affect our ability to detect panel or time effects on the microclimate factors measured, and all data were included in the analysis. The data were then aggregated by hour and day of year. A repeated measures ANOVA was used to test the effects of panel influence, time (days within a season), site, and their interaction on relative humidity and air temperature for each season and diurnal period. Soil temperature data were treated equivalently, but analyzed with a mixed model, with block treated as a random effect. All statistical analyses were performed in RStudio (version 1.0.153; R version 3.5.1 [2018‐07‐02]).

**Figure 5:**
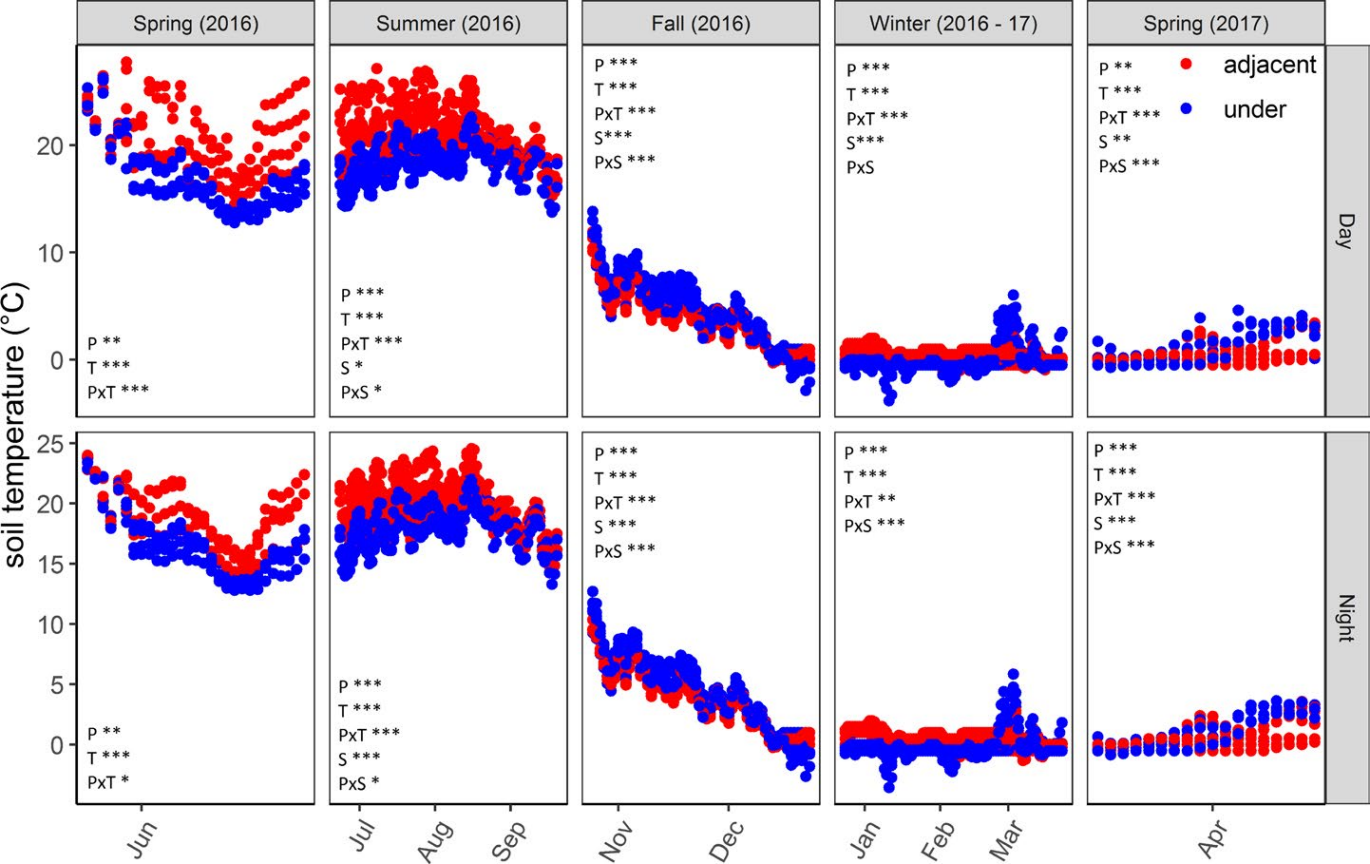
Soil temperature trends from March 2016 through February 2017. A mixed‐model repeated measures ANOVA for each season is presented. P = panel effect, T = day of year effect, PxT = interaction between panel and time, S = site effect, PxS = interaction between site and panel.

Notably, unlike more hot and arid PV array sites (Barron‐Gafford et al., 2016), we did not detect a heat island effect of the arrays; despite the marked effects of the panel on soil temperature, no significant panel effect on air temperature or relative humidity was detected in fall (2016), winter (2016–2017), or spring (2017) at any of the three sites. There was a marginally significant increase in relative humidity (*F* = 3.3, *P* = 0.07) and reduction in air temperature (*F* = 3.1, *P* = 0.08) under the array panels in the spring (2016), but no significant site interaction or site‐by‐panel interaction was detected. In contrast to air temperature and relative humidity, there was a clear panel effect on soil temperature at 10‐cm depth across all sites, seasons, and diurnal periods (*P* < 0.01 in all cases) and site‐by‐panel interactions in all seasons except spring (2016) (*P* < 0.05 in all cases) (Fig. 5).

In late spring and summer, the A soil was warmer than U at all sites, which correlates with greater PAR exposure. The U and A soil temperature began converging in the late summer and into the fall as the difference in PAR between the U and A areas declined. In the fall, U areas had warmer soil temperatures until the first major snowfall event, when A became warmer than U, likely reflecting snow insulation of the A soil relative to the precipitation‐intercepted U areas (Monson et al., 2006). In the winter, A areas tended to be warmer than those beneath the panels because of increased snow pack. Surprisingly, this effect was reversed at the Vermont Agricultural Center, which had consistently warmer soil in the U areas despite less snow cover, a pattern that may reflect higher soil moisture in the U areas supplied by subterranean water flow.

Soil decomposer activities over the winter are a critical regulator of plant‐available nutrients following thaw (Aanderud et al., 2013); therefore, changes to winter soil temperature regimes within an array site (Fig. 5) may be particularly relevant to the plant productivity and community dynamics during the growing season. A suite of other biological changes observed in the U versus A areas in our array study sites (Sistla et al., unpublished data) suggest that these microhabitat changes that affect soil temperature but are decoupled from comparable changes in air temperature can promote cascading effects on the plant–soil system even relatively shortly after a change in land use such as array development.

## DISCUSSION

### Diverse applications of iButtons

Although iButtons were designed for commercial applications related to product management (and now number 175 million in circulation), the availability of these relatively inexpensive data loggers has inadvertently summoned a wave of novel research in such disciplines as ecology, physiology, and earth science. Several comparable products are commercially available, such as the TidbiT (Onset Computer Corporation), Atmos 41 (Meter Group, Pullman, Washington, USA), and iButton Thermochron temperature data loggers and Hygrochron temperature and humidity data loggers from Maxim Integrated. A number of iButton models are available, optimized for different applications, with different temperature ranges, accuracy, resolution, and sampling rates, and prices vary accordingly. Their accuracy, well within the ±1·0°C claimed by the manufacturer, has been upheld by independent assessment (Hubbart et al., 2005), with improved resolution available for certain models. For applications where air temperature measurements are required, solar radiation shields control for direct heating of the sensor by sunlight. For a thorough review of commercially available radiation shields, see Hubbart (2005), and for an inexpensive alternative, see Hubbart (2011). Mittra et al. (2013) provide a helpful step‐by‐step technical guide to using iButtons in the field.

Biological applications of this technology include the study of animal behavioral physiology (Brower et al., 2008; Thompson et al., 2016), hibernation (Rasmussen and Litzgus, 2010; Vanderwolf et al., 2012; Zervanos et al., 2013), and thermal tolerance (Denny et al., 2011). Plant ecologists have employed iButtons to characterize extreme microclimates (Chambers and Emery, 2016), which can then inform experimental design. Understanding the complex and nuanced thermal environment experienced at or below the soil surface can illuminate mechanisms underpinning species interactions, because microclimatic conditions are an important regulator of whether species facilitate or compete with one another (Spasojevic et al., 2014; Copeland and Harrison, 2017).

Data loggers also have broad applications in the earth sciences and have been used to make precise measurements of snow accumulation and melt (Lundquist and Lott, 2008), which has major implications for plant life at high latitudes and elevations. Hydrologists have used iButtons to gauge groundwater and surface water interactions (Naranjo and Turcotte, 2015), which could improve our understanding of riparian vegetation ecology, and the dynamics of the rhizosphere more generally. We demonstrate their utility for identifying and tracking the development of novel microhabitats with land use changes resulting from the built environment and agricultural areas, which may facilitate unique plant assemblages by affecting organic matter decomposition and moisture availability.

In addition to the myriad applications of iButtons and similar sensors for the field botanist, they also have utility in experimental laboratory settings. Greenhouses are notorious for varying microclimatic conditions, which can be a major source of unwanted variation in experiments and may negatively impact crop production. Deployment of data loggers can help greenhouse managers first document, then optimize growing conditions throughout the greenhouse (Kutta and Hubbart, 2014; Vallone et al., 2017).

### Experimental considerations

A major benefit of iButtons over previous technology is their compact size and relative affordability. This enables data to be collected inconspicuously in areas frequented by humans or other animals. In southeastern Turkey, our sites are exposed and used by local shepherds, thus making them unsuitable for standard soil moisture loggers, as they would almost certainly be removed by any curious passersby. Because of this, we did not use PVC pipes or other protections and obvious markings to assist with iButton recovery. Unfortunately, our low recovery rate in Turkey was exacerbated by inadequate marking of microsite placements. A few of the sites used for the rock fern study were popular hiking trails. Although in most cases every effort was made to obscure the iButton, they were sometimes discovered. We included contact information, written in permanent marker on the PVC housing, and it resulted in the return of one iButton that apparently became dislodged. Stopping short of elaborate and potentially destructive restraints, preventing iButton loss to raccoons proves more challenging.

For the chickpea study, we used Hygrochron data loggers with the expectation that they would provide correlative data on soil moisture we could use from relative humidity estimates. Wild *Cicer* grows in rock crevices and other complex habitats where soil moisture is particularly difficult to measure but likely quite important to the plant. However, during the rainy Mediterranean winter, the Hygrochrons failed to accurately record relative humidity while soils were saturated, a design limitation of the technology. Furthermore, we did not find a significant correlation with soil moisture. In the rock fern study, we mounted the Hygrochrons within a small section of PVC pipe that protected them from direct precipitation and allowed them to drain, thereby avoiding saturation of the sensor. Nevertheless, we experienced a 65% failure rate of iButton Hygrochrons after a one‐year deployment.

Although iButton Thermochrons are designed to be waterproof, failures have been noted (Wolaver and Sharp, 2007), especially when submerged for long periods or at depth. For applications in saturated soils, or underwater, a waterproof iButton capsule (model DS9107) is available from the manufacturer. Another effective and affordable waterproofing option is Plasti Dip (Plasti Dip International, Blaine, Minnesota, USA) (Lundquist and Lott, 2008), although it should be noted that this coating has a minor influence on temperature readings (Roznik and Alford, 2012). Securing buried iButton Thermochrons in conical tubes proved to minimize data logger failure and loss. Unfortunately, iButton Hygrochrons are apparently much more susceptible to failure in humid or wet conditions (Vanderwolf et al., 2012), possibly because of the humidity sensor aperture, which cannot be covered. Because of this, use of unprotected iButtons is not recommended in settings that may experience saturated conditions or flooding. Therefore, the benefit of recording both temperature and humidity data with a single device should be weighed against the risk of Hygrochron failure in the absence of waterproofing options available for the Thermochron, and the unclear relationship of soil relative humidity to water available for plant growth. Although the Hygrochron function may be tempting to use as a proxy for measuring soil moisture, we advocate that this application be used cautiously, if at all.

### Conclusions

Temperature and humidity data loggers such as iButtons are effective tools for assessing fine‐scale climatic variation in wildland settings, agricultural areas, and the built environment. We suggest they may be utilized for improved parameterization of phenological models, which may contribute to agricultural improvements. We demonstrate their utility for characterizing fine‐scale effects of anthropogenic structures on soil temperatures, providing the foundation for a more nuanced understanding of the ecological ramifications of the built environment. We show that iButtons can play an important role in capturing microclimate conditions that may be associated with refugia, a major limitation of most climate models. By incorporating the influence of fine‐scale topography and hydrology, the accuracy of climate models will greatly improve, and empirical measurements from data loggers such as iButtons can play an important role in model validation. Finally, areas that include rock outcrops or other features associated with mesic microclimates deserve careful consideration for conservation prioritization, as these areas may provide microrefugia that buffer species against climate change.

## Data Availability

Code and data for the chickpea study are available at: https://github.com/ericvonwettberg/iButtons.
